# A Practical Treatise on Medical Jurisprudence, with so Much of Anatomy, Physiology, Pathology, and the Practice of Medicine and Surgery, as Are Essential to Be Known by Members of Parliament, Lawyers, Coroners, Magistrates, &c.

**Published:** 1834-10-01

**Authors:** 


*A Practical Treatise on Medical Jurisprudence, with so much of Anatomy, Physiology, Pathology, and the Practice of Medicine and Surgery, as are essential to be known by Members of Parliament, Lawyers, Coroners, Magistrates, Officers in the Army and Navy, and Private Gentlemen; and all the Laws relating to Medical Practitioners. With Explanatory Plates.* By J. CHITTY, Esq., Barrister at Law.—London, 1834. 8vo. pp. 466.

THE general object of works on legal medicine is to illustrate those points on which law and medicine become so blended, that knowledge derived from the studies peculiar to each faculty is requisite for their elucidation. Several excellent works of this kind already exist, to which Mr. Chitty has it in view to add another, whose plan is in a great measure new. Our author, justly considering that much of the difficulty in which medical jurisprudence has been involved arises from jurists being for the most part very ignorant of medicine, and medical practitioners equally ignorant of law, proposes to remedy this evil by supplying the two professions with such a summary of each other's science as may put them respectively in a condition to meet on terms of better understanding. The idea is a good one, but we fear Mr. Chitty is performing his task on far too large a scale.

This first volume, devoted entirely to anatomy and physiology, contains a huge, though not very well compacted, mass of information on the subjects of which it treats: it embraces, however, more knowledge than is either necessary or attainable for one who does not make medicine his profession. If the lawyers can digest it all, we wish them joy; but we sincerely hope that, when Mr. Chitty prepares his law-feast for the doctors, his hospitality may be less profuse, or we are apprehensive that our feeble powers of assimilation will not enable us to do it justice. It appears to us that our author proceeds too much on march-of-intellect principles with reference to the diffusion of medical and legal knowledge. "A general knowledge of these subjects," he says, "is essential to the perfect education of all officers in the army and navy, and of every gentleman, and especially so to legislators, judges, coroners, magistrates, barristers, and indeed to every member of the legal profession, and to all persons who may become jurors or witnesses; so as to enable legislators more scientifically and practically to determine upon the expediency of improving the existing laws relating to public health, and the protection of the persons of individuals, and also to give due effect to existing regulations."

And again,

"With respect to every private gentleman, we shall only repeat the just observation of a powerful advocate for extended education, that in the present day a knowledge of physics, or natural philosophy in general, and in particular of physiology and pathology, is indispensable not merely to all persons engaged in scientific pursuits, but to all who pretend to a moderately good education. To all country gentlemen in particular, many of whom reside at a considerable distance from any medical practitioner, it will be highly important to be able, on emergencies, either to direct or assist in recovering a person in suspended animation, or in reducing a dislocation, or in stopping hemorrhage, or performing numerous other acts, as hereafter pointed out; or at least to assist a medical practitioner in many of his endeavours. In the country, some at least of the medicines and apparatus usually required on emergencies should be kept in each gentleman's house, and from time to time renovated, so as to be ready for immediate application by the medical adviser, in case of accident or sudden illness, as the saving even of life may frequently depend on the promptness of the remedy: a general knowledge of the outline of these subjects would enable heads of families to afford, in many cases, immediate and effectual relief, and at all events would enable them more distinctly to explain to the medical adviser antecedent symptoms and appearances, and to understand and apply his directions, and thereby greatly to contribute to the cure and comfort of every member of his family. So, independently of accidents and sudden occasion for assistance, the instances are innumerable in which unprofessional but well-informed individuals may greatly alleviate the sufferings of their fellow-creatures; as, by suggesting various instruments or means of cure, or at least of mitigating pain or annoyance, wholly independent of medicine,—as the use of the hydrostatic bed for an invalid, or the use of various other discoveries, at present wholly unknown to the bulk of society: and surely no one who can anticipate the gratification incident to relieving a fellow-creature in danger or pain should refrain from cultivating such useful and important knowledge." (*Preface*, p. xiii.)

Now we decidedly hold all knowledge to be excellent, and think that every man should know everything, *if he could;* but the misfortune is, that one individual can only have a certain quantity of knowledge; and if any man is master of one difficult branch, to which he particularly addicts himself, and has sufficient acquaintance with others to enable him to think and converse rationally on them in a very general way, we conceive him to be an exceedingly well-informed person, and much superior to the majority of his species.

We leave it to the jurists to determine what would be the effect of every client's having "some knowledge" of the practice of law; but, for our own parts, we are always bored to death with those patients who "have studied medicine a little," and find that such patients are longer in getting cured than anybody else.

To return to our subject—the relation of law and medicine to each other. It appears to us that the professors of these two sciences do not require anything like an extensive acquaintance with each other's departments: and it is fortunate they do not, for the acquisition would be impossible to men of ordinary capabilities. There are, nevertheless, certain points on which it is highly necessary that they should be mutually informed; and here, as in all other cases, knowledge, to be available, must be *perfectly accurate* as far as it goes.

The medical information essential to the jurist relates only to certain facts, which are neither very numerous nor very difficult to be learned or remembered; with the theoretical part of the science he need have nothing to do. We even think that an acquaintance with medical opinions and reasonings, so far from assisting a counsel in putting questions to a witness, would generally be a disadvantage to him, as it would render him liable to bewilder both the witness and himself, by getting entangled in theories, which, if they have once taken possession of his mind, he will not always find it easy to separate from the facts. The medical practitioner is in no danger of confounding the two, because the facts consist of what he *sees* and *does*, the theories merely of what he thinks about: but it is otherwise with the jurist, since nothing with him is matter of experience. We should say, therefore, that an accurate acquaintance with a certain number of medical facts, and such an idea of the general scope of the science as may prevent these from being absolutely bald and insulated, is all that is needful for the practitioner of law. In like manner, the legal knowledge of a medical man should, we conceive, be characterised rather by accuracy than extent: there are comparatively few judicial points which demand the aid of medicine for their illustration, but on these the practitioner should be perfectly well informed, in order that he may perceive the precise bearing of the evidence he is to give; since an answer, though truly and correctly given, may afford little useful information, from the respondent's not rightly understanding the view with which the question was asked. It is, of course, also incumbent on medical practitioners to know thoroughly the laws relative to their own profession,—its privileges, immunities, and liabilities.

In giving an opinion on Mr. Chitty's book, we may affirm that, although its object appears to us not altogether attainable, from its too great extent, it is nevertheless likely to prove of great utility to both the professions for whose use it is intended. The Part before us, as we have already mentioned, contains a vast deal of anatomical and physiological information; and although, if it proceeded from the pen of one of our own brethren, we might pronounce it somewhat ill assorted, and chaotic withal, we have much pleasure in bearing testimony to its general accuracy, which the author has endeavoured to ensure by a laborious reference to the best authorities.

This volume is a curiosity of its kind: from the peculiar circumstances in which the author is placed, in compiling from such various sources an extensive treatise on subjects with which he is not himself practically familiar, the work presents the aspect of a literary mosaic, in which the numerous pieces of which it is composed, though carefully placed in juxta-position, may easily be perceived not to form a continuous whole: *e. g.*

"In the extremities the veins consist of two sets, one running immediately under the skin, termed subcutaneous, and the other deep-seated, accompanying the arteries. The anastomoses or in-osculations of the veins are greater and more frequent than in the arteries; those of the veins are by large branches, whilst the arteries inosculate generally by small.*

"The veins rarely, if ever, ossify like the arteries,† but they are subject to several diseases, as inflammation, relaxation, tumours, ruptures, &c.‡ Veins also are subject to varix, and, when affected by it, are called varicose. The term varix is to veins a disease, what the true or encysted aneurism is to arteries. It is applied by surgeons to the permanently dilated state of a vein attended with an accumulation of dark-coloured blood, the circulation of which is materially retarded in the affected vessels. When veins are varicose, they are not only dilated, but they are also evidently elongated; for, besides being irregular, and in several places studded with knots, they make a variety of windings, and, coiling themselves, form actual tumours. Varices are most commonly observed in the lower extremities, reaching sometimes even as far up as the abdomen. They have, however, been noticed in the upper extremities, and it is probable that the whole venous system is susceptible of the affection. As a well-informed writer observes, the great venous trunks sometimes become varicose. When the disease is situated near the heart, it is attended with pulsation, which renders it liable to be mistaken for aneurism.§

"Veins are liable to ruptures, wounds, and laceration. Hemorrhage from veins, though not so dangerous as from arteries, is yet attended with danger.||

"The enumeration of each of the three thousand veins, even if practicable, would occupy more space than can here be afforded, and therefore we can only refer to the particular authors who have written upon them distinctly.¶ We have seen that they have been estimated at even three thousand, but the most intelligent anatomists do not distinguish by name even two hundred."** (P. 147.)

The work, however, is a meritorious one, and we wish the author all success in its prosecution.

* "Coop. Dict. tit. Anastomosis, 110, and tit. Inosculation, id. 761; Tuson's Comp. 126; Quain's El.

† "2 Good, 14; 5 Good, 360.

‡ "See in general Hodgson on Diseases of Arteries and Veins; Arnott's Med. Chir. Trans. vol. xv. 47; Coop. Dict. tit. Veins, diseases of, 1206.

§ "See further Coop. Dict. tit. Varicose Veins, 1202; 3 Good, 476.

|| "Coop. Dict. tit. Hemorrhage, 625; 3 Good, 183, as to hemorrhage.

¶ "See enumeration, Tuson's Comp. 126 to 136; 2 Bell, 307 to 321; Lizars, parts ii. iii.; Quain's El. 274 to 279, 366, 411 to 413, 485, 486, 588, 589; see plate and enumerations, 3 Gregory's El. Nature, 255 to 261.

** "See the names of the principal veins, and their situations, 3 Gregory's El. Nature, 261."

